# Supplementary material for: Lactoferrin Induces the Synthesis of Vitamin B6 and Protects HUVEC Functions by Activating PDXP and the PI3K/AKT/ERK1/2 Pathway
Source: Int J Mol Sci. 2019 Jan 30;20(3):587. doi: 10.3390/ijms20030587 (PMC6387185; doi:10.3390/ijms20030587)
Supplement: Supplementary file 1 [file ijms-20-00587-s001.zip › Supplementary table 1 in venn results.pdf]

Table S1. Screened genes

**Group**	R1	R2	R3	R4	R5	R6
**Description**	C vs H down	C vs H up	C vs L down	C vs L up	L vs H down	L vs H up
**Screened genes**	PTGES3L-AARSD1	ARL6IP6	MT-ATP6	MBNL1	AC084117.1	AC121761.1
	RPS21P1	ZBTB14	PARD6G-AS1	DDX28	HILPDA	U62317.3
	AL031710.2	ZNF510	AL390726.5	AL035425.3	ADM	HLA-F-AS1
		EID2	AC008770.1	SAP30	SLC2A3	LGALS1
		ETAA1	SH3BP1	PKIA	AC010655.4	AL136982.4
		ZNF24	AP000346.2	CFAP97	PCBP2-OT1	G0S2
		OTUD6B-AS1	GUCA1B	ZNF292	CITED2	F11R
		MAFB	MYC	KDELC2	AC005363.1	MTATP6P1
		VIM-AS1	ZNF710-AS1	HOXA5	PLIN2	AC069499.1
		TRIQK	AC234775.3	TIPARP	SPRY1	AL391244.3
		PBX1	AC021097.1	FLJ37453	NAMPTP1	MTND2P28
		AF106564.1	AC011445.1	RLF	CXorf40A	MT-ATP8
		PRMT6	AC136475.2	C11orf96	LOX	MT-ND3
		SFT2D3	RPS10-NUDT3	ARRDC3	PEX2	AC232271.1
		TIGD5	RPS18P9	OMA1	KDM3A	DHRS2
		C7orf25	MORF4L2-AS1	AC083843.2	AC008115.3	AL355987.3
		PDXP	TAOK2	FOXC1	SMG1P1	KCNK9
			RBM14	AC006064.4	"TSPAN14"	AL110118.2
			LINC01311	ZIC5	VEGFA	
			KIAA0391	ARFIP1	DDIT3	
				FOXD1	GOLGA6L10	
				HOXA11	LDHAP4	
				DLX2	AC073046.1	
				RNF128	SNHG25	
				ZNF518B	P4HA1	
				AC009948.1	CSRNP1	
				KC877982.1	ATF3	
				NT5DC1	SMG1P3	
				ID2	GINS4	
				INSM1	NHSL1	
				AL035425.2	SLC7A5P2	
				FIBIN	FGF9	
				KRCC1	GADD45G	
				AP000295.1		
				AC004080.3		
				AC004080.5		
				DDIT4-AS1		
				PDXP		
